# Detecting the Sigma Phase in Duplex Stainless Steel by Magnetic Noise and First Harmonic Analysis

**DOI:** 10.3390/ma17184561

**Published:** 2024-09-17

**Authors:** João Silva, Edgard Silva, Augusto Sampaio, Rayssa Lins, Josinaldo Leite, Victor Albuquerque Silva, João Manuel R. S. Tavares

**Affiliations:** 1 Academic Unit of Industry, Federal Institute of Education, Science and Technology of Paraíba, Av. Primeiro de Maio, 720—Jaguaribe, João Pessoa 58015-435, Brazil; silvajbo@ifpb.edu.br (J.S.); edgard@ifpb.edu.br (E.S.); augusto.sampaio@academico.ifpb.edu.br (A.S.); rayssasatlins@gmail.com (R.L.); 2 Department of Mechanical Engineering, Federal University of Paraíba, Jardim Universitário, s/n—Castelo Branco, João Pessoa 58051-900, Brazil; josinaldo@ct.ufpb.br; 3 Department of Teleinformatics Engineering, Federal University of Ceará (UFC), Fortaleza 60455-970, Brazil; victor.albuquerque@ieee.org; 4 Instituto de Ciência e Inovação em Engenharia Mecânica e Engenharia Industrial, Departamento de Engenharia Mecânica, Faculdade de Engenharia, Universidade do Porto, Rua Dr. Roberto Frias, S/N, 4200-465 Porto, Portugal

**Keywords:** magnetic noise, non-destructive test, duplex stainless steel

## Abstract

Non-destructive electromagnetic tests based on magnetic noise analysis have been developed to study, among others, residual stress, heat treatment outcomes, and harmful microstructures in terms of toughness. When subjected to thermal cycles above 550 °C, duplex stainless steels form an extremely hard and chromium-rich constituent that, if it is superior to 5%, compromises the steel’s corrosion resistance and toughness. In the present work, a study was carried out concerning the interaction of excitation waves with duplex stainless steel. Hence, by analyzing the magnetic noise and variations in the amplitude of the first harmonic of the excitation waves, the detection of the deleterious sigma phase in SAF 2205 steel is studied. To simplify the test, a Hall effect sensor replaced the pick-up coil placed on the opposite surface of the excitation coil. Sinusoidal excitation waves of 5 Hz and 25 Hz with amplitudes ranging from 0.25 V to 9 V were applied to samples with different amounts of the sigma phase, and the microstructures were characterized by scanning electron microscopy. The results show that the best testing condition consists of applying waves with amplitudes from 1 V to 2 V and using the first harmonic amplitude. Thus, the test proved effective for detecting the formation of the deleterious sigma phase and can follow the ability to absorb energy by impact and, thus, the material embrittlement.

## 1. Introduction

Duplex stainless steels are characterized by presenting equal volumetric percentages of ferrite and austenite constituents in their structure [1,2,3]. However, when thermal cycles above 550 °C are allowed, a harmful phase called sigma is formed, with a hardness of around 900 HV and rich in chromium. The high hardness compromises the energy absorption capacity by impact, i.e., toughness, and the high chromium content reduces the corrosion resistance [4,5,6,7]. The paramagnetic sigma phase arises from the ferrite structure, which is ferromagnetic, changing the material permeability. However, it does not occur in the austenite phase, which is paramagnetic [1,3,4,6].

Low quantities of the sigma phase promote a considerable decrease in toughness without a notable influence on the hardness. For instance, the precipitation of 1.3% of this phase decreased the impact toughness from 320 J (solute-treated) to 24 J (aged samples at 800 °C for 10 min) [1,2,4,5], and, as the precipitation of the sigma phase increases, cracks occur preferentially in the contours of the particles of this phase. Also, as the sigma phase is rich in chromium, it deflects the adjacent region of Cr, reducing corrosion resistance [1,5].

Changes in magnetic permeability characterize the formation of new constituents by phase transformations in ferromagnetic materials. This makes non-destructive electromagnetic tests interesting, such as the ones based on eddy currents, magnetic Barkhausen noise (MBN), and magnetic permeability measurements in the reversibility region of the movement of magnetic domain walls. These tests have been applied, for example, to studies of heat treatments, residual stress, fatigue, the presence of second phases, the integrity of welded joints, and embrittlement [2,3,8,9,10,11,12,13,14,15,16,17,18,19].

The imposition of an electromagnetic wave on ferromagnetic materials leads to the movement of the magnetic domain walls and an interaction of these with the material’s microstructure. Factors such as grain boundaries, grain size, precipitates, dislocations, second-phase particles, and residual stresses act as anchorage points for the movement of the walls, and when the external magnetic field strength is sufficient for them to overcome the obstacles of the microstructure, the generation of magnetic Barkhausen noise occurs. This noise analysis brings information about the changes in the material, which is valuable in non-destructive electromagnetic tests [20,21,22,23,24,25,26,27,28,29,30,31,32,33,34,35].

Electromagnetic tests based on MBN are characterized by two coils: the excitation, which applies the signal, and the pick-up, which acquires the interaction signal. In these, there are three configurations of pick-up coils: one on the same surface as the excitation, another on the opposite surface, and the last surrounding the material. From these, the most common is the layout on the same surface of the excitation coil. A pick-up coil wound closely around the sample has also been used in studies of stress measurements in thin film samples, where the thicknesses are relatively small [16]. These tests have various configurations for the devices used, mainly in terms of excitation coils and receivers, the shape of the cores of the coils, and the characteristics of the signal applied to them, such as in terms of amplitude, frequency, and type of wave. Positioning the receiver coil on the opposite surface favors studying along the thickness of the material, thus sweeping a larger volume and allowing the evaluation of areas thermally affected by processes such as welding. Positioning the excitation coil on the same surface is limited in this regard, and the surrounding coil requires different configurations depending on the thickness of the material to be analyzed [10,13,15].

When a magnetic field is applied to a ferromagnetic material, the resulting shape of the magnetic induction is distorted due to magnetic hysteresis, and the non-linearity of the material’s permeability, i.e., a sinusoidal shape wave being applied induces a non-sinusoidal one. This distorted magnetic induction waveform contains components at the harmonic frequencies of the applied magnetic field [23,24,26]. Harmonic analysis has been applied to investigate material failures, detect corrosion degradation, perform non-destructive evaluations of steels subjected to heat treatment, and monitor harmful microstructures in stainless steels [23,24,25,26,27,28].

Electromagnetic tests in the reversibility region of the magnetic domain walls’ movement have used Hall effect sensors to detect the interaction of magnetic flux density and the material and, thus, analyze microstructural variations, magnetic anisotropy, and residual stress, among other applications. These sensors are sensitive to small variations in the intensity of the magnetic flux passing through a material, cost only a few dollars, and are simple to use. There are initial studies for replacing the receiver coil in tests in the irreversibility region. However, a deeper study of the applied wave intensity and a better transmission wave frequency is lacking in the literature [2,3,8,10].

Magnetic permeability measurements using a Hall effect sensor have proven effective for studying the α’ phase in duplex stainless steels. The results confirm that Hall voltage measurements are affected by the phase transformations that occur in SAF 2205 duplex stainless steel at 425 °C and 475 °C and are suitable for tracking the formation of the α´ phase in a non-destructive way. This was confirmed by correlation with the X-ray diffraction technique, an already well-consolidated inspection technique [3]. Similar results were obtained by authors who observed a decrease in magnetic susceptibility measurement in the same temperature range [29]. The microstructure formed prevents the movement of the magnetic domain walls, and, therefore, the magnetic susceptibility is decreased.

Permeability measurements have also been used to detect the formation of the sigma phase in the temperature range from 700 °C to 1000 °C through measurements carried out with Hall effect sensors and applying magnetic field strengths of up to 300 A/m in a pick-up coil. An ideal external magnetic field should be applied to obtain the best amplitude for the phase assessment. A value of 211.5 A/m has been suggested as the ideal field. The paramagnetic sigma phase reduces the induced magnetic field even when the phase is in reduced quantities of 2% [2]. The sigma phase was also studied in SAF 2205 duplex stainless steels at temperatures of 800 °C and 900 °C by analyzing the MBN with emitting and receiving coils positioned on the same surface and applying 10 Hz sinusoidal waves. Root mean square (RMS) values were correlated with the amount of the sigma phase. The MBN intensity was significantly reduced with the increased heat treatment time, indicating fewer ferromagnetic phases [4,7].

MBN noise has been applied to analyze several types of materials. The effect of quenching embrittlement in supermartensitic steels was studied in samples treated at temperatures of 620 °C and 640 °C cooled in water and in an oven after tempering, and those cooled slowly showed a lower toughness and a higher volumetric fraction of austenite [10]. Another study analyzed the stress profile generated in a 1070 steel sample subjected to a three-point bending test. The results suggested that the technique can detect the applied voltage profiles. Furthermore, the variation in microstructure in carbon steel welded joints was monitored by MBN. The results permitted the identification of the welded joint’s heat-affected zone (HAZ) using MBN signals [30].

Non-destructive inspection techniques based on MBN have also been applied to detect non-homogeneous regions in carbon steel sheets. The non-homogeneous or damaged regions were produced by plastic deformations in rolled and annealed sheets of SAE 1060 and 1070 steels. The behavior of the mean square root of the Barkhausen magnetic noise signal was correlated with the position of the uneven regions detected in the samples. The results showed that in all the studied cases, it was possible to detect the position of the damage through the variation in the magnetic field [31]. Another application based on MBN is detecting early-stage fatigue, which is associated with plastic deformation in ferromagnetic metallic structures. Experimental results demonstrated that MBN is promising for this characterization [32]. The presentation of the behavior of the interaction between excitation waves and different types of anchoring points, presented previously, serves as a foundation for understanding the influence of the microstructure on magnetic noise.

In the present work, a study was carried out of the best excitation wave to be applied in duplex stainless steel (DSS) to detect the formation of the sigma phase in a configuration where the receiving coil, positioned on the opposite surface to the excitation, is replaced by a Hall effect sensor to simplify the test. In this, the magnetic noise and the harmonic of the emitting wave were studied, as well as their ability to follow the formation of the harmful sigma phase. The methodology presented was applied to duplex stainless steel because, under the studied condition, only one harmful constituent is formed from the decomposition of ferrite. This would facilitate the interpretation of the signals resulting from the interaction between the excitation waves and the material, as well as the analysis of the methodology. Furthermore, the applied field strength was carried out in the region of magnetic reversibility of the movement of the magnetic domain walls [2,3] and the magnetic noise analyzed, which, according to the literature, will not be termed Barkhausen, as it is not in the irreversibility region.

## 2. Materials and Methods

The material studied in this work was SAF 2205 hot rolled steel supplied in plate format with a thickness of 8 mm. To carry out the experiments, four duplex stainless steel SAF 2205 samples were used, which were machined by electro-erosion in a circular shape with a diameter of 24 mm, one without aging, and three underwent thermal treatments at a temperature of 850 °C for ¼ h, 1 h, and 2 h in a resistance oven and were then cooled in water. The treatment at 850 °C for ¼ h can generate 5% of the sigma phase in the material, which is enough for the steel under study to become embrittled, affecting its microstructure and compromising its mechanical properties [1,2,3]. The other treatment times are sufficient to consolidate the formation of the sigma phase and serve to follow the deleterious sigma phase.

The plate was received in the hot rolled state, and all the measurements were performed in the rolled direction, corresponding to the easy magnetic direction. The direction of easy magnetization of the received material was determined by the non-destructive technique for measuring magnetic anisotropy developed by [8]. The procedure used to study microstructural anisotropy was a non-destructive test carried out in the region of magnetic reversibility using direct current. It consisted of applying a magnetic flux intensity in the center of a circular sample 24 mm in diameter and 8 mm in thickness to determine the magnetic flux density resulting from the interaction through a Hall effect sensor applying a frequency of 5 Hz and 10 V. The sample was rotated from 0° to 360° in 22.5° steps.

Duplex stainless steel was chosen for this study because it is widely used in industry due to its toughness and corrosion resistance characteristics in the as-received condition. However, when subjected to thermal cycles, such as in the welding process, the sigma phase may form from the decomposition of the ferromagnetic ferrite phase. A mere 5% of the sigma phase is enough to compromise its mechanical properties and corrosion resistance, as this phase has a hardness of around 1000 HV and is rich in chromium, thus impoverishing its matrix and reducing its resistance to corrosion.

Manufacturing processes of the studied steel, such as welding, require non-destructive testing to evaluate their quality by detecting the formation of the sigma phase and predicting corrective interventions. The apparatus used in this study can be applied to detect the presence of this harmful constituent. Furthermore, in the studied temperature range, the steel under analysis presents only the formation of a harmful constituent to its properties, making it easier to predict the origin of the changes in the used signals.

The samples were prepared for optical and scanning electron microscopy analysis, the first to detect the amount of the sigma phase and the second for the microstructural analysis. The samples were maintained under chemical attack with an electrolytic solution of 10% KOH using a voltage of 3.7 V and a current of 0.75 A for ¼ h, which preferably reveals the sigma phase and facilitates its visualization and identification, i.e., segmentation. It is possible that some χ phase was also formed and quantified as σ phase. However, it was not the objective of this work to separate these two phases because they have similar effects on steel’s properties. The χ phase forms itself before the precipitation of the σ phase and disappears once the σ phase starts to precipitate [1]. Twenty images of each condition were acquired and segmented. The amount of the constituent sigma phase was determined according to a 95% confidence interval.

Ferrite microhardness measurements in the as-received and treated for ¼ h and 2 h conditions were carried out to analyze the presence of the sigma phase. Ten measurements were taken for each condition, and the confidence interval was obtained at 95%. A Shimadzu microhardness tester model HMV-G 20 S was used with an applied load of 250 g.

The X-ray diffraction tests were carried out using copper Kα radiation, with a voltage of 40 kV and a current of 30 mA; a step of 0.02°, with a time per step equal to 9.6 s; and adopting a viewing angle (2θ) ranging from 41° to 53°.

**Figure 1:**
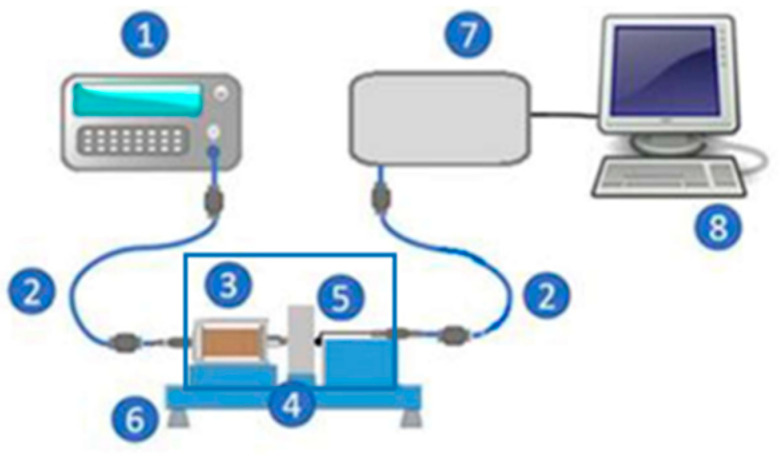
Used experimental setup: (1) signal generator, (2) shielded cables, (3) excitation coil, (4) material sample, (5) Hall effect sensor, (6) test bench with Faraday cage, (7) acquisition board, and (8) computer.

The workbench consists of two modules, one for excitation and the other for acquisition. The scheme of the experimental setup is shown in Figure 1.

The excitation module comprises a function generator (Minipa MFG 4205B model, São Paulo, Brazil) and a transmitter coil. The function generator transmits waves of different formats to the pick-up coil. Here, a sinusoidal wave was chosen since it presents the least interference from the harmonics of the excitation wave. The coil was positioned in the center of one of the sample faces, inducing a magnetic flux density in the material. The studied waves were applied to the center of the circular samples, and the signal resulting from the interaction was acquired on the opposite surface. The surfaces of the samples were sanded with 320-grit sandpaper to eliminate possible surface oxidation during cooling after treatment.

The acquisition module comprises a Hall effect sensor, an acquisition board, and a computer. The sensor is positioned in the center of the other face of the sample to detect the interaction field of the interaction between the excitation wave and the material. The acquisition board connects to the sensor and the computer via USB cables. The computer performs automatic data acquisition using software developed in-house. The chosen SS495A model is a Hall linear effect sensor with a sensitivity of 3.125 mV/gauss and an input voltage between 0 V and 10 V, it being supplied here with a continuous voltage of 5 V. This sensor has a working range of up to 700 gauss, and the measurements were carried out at around 550 gauss, remaining below its saturation region.

To detect the characteristics of the excitation waves that can follow the sigma phase formation, the as-received and treated at 850 °C for ¼ h samples were analyzed. Thus, the characteristics of the wave station that can have the sensitivity to detect the presence of 5% of the sigma phase from the thermal treatment were determined. The best condition of this analysis was then used to follow the formation of this harmful phase in samples with different amounts of sigma phase, mainly corresponding to ¼ h, 1 h, and 2 h of treatment times, as well as in the initial condition.

The signal analysis was divided into two stages. In the first one, high-pass filters for magnetic noise analysis were applied. Thus, using a fast Fourier-transform (FFT) algorithm, the signals were transformed to the frequency domain and then processed by 50 Hz and 250 Hz digital high-pass filters. These cutoff frequencies were used to analyze the noise region. The second stage involved analyzing the harmonics of the 5 Hz and 25 Hz excitation waves by identifying their peaks based on the signal’s root mean square (RMS).

Thus, based on the signal’s RMS parameter analysis, the best condition was used to follow the sigma phase formation in the SAF 2205 material. The excitation voltages of the waves used were 0.25 V, 0.5 V, 0.75 V, 1 V, 1.25 V, 1.5 V, 1.75 V, 2 V, 3 V, 5 V, 7 V, and 9 V. These values refer to the magnetic field strength applied to the samples of 1.4 Oe, 2.3 Oe, 3.4 Oe, 3.9 Oe, 4.6 Oe, 5.2 Oe, 5, 6 Oe, 6.7 Oe, 9.9 Oe, 15.7 Oe, 22.4 Oe, and 28.2 Oe, respectively. For each condition, 50 measurements were taken, and the 95% confidence interval was determined.

**Figure 2:**
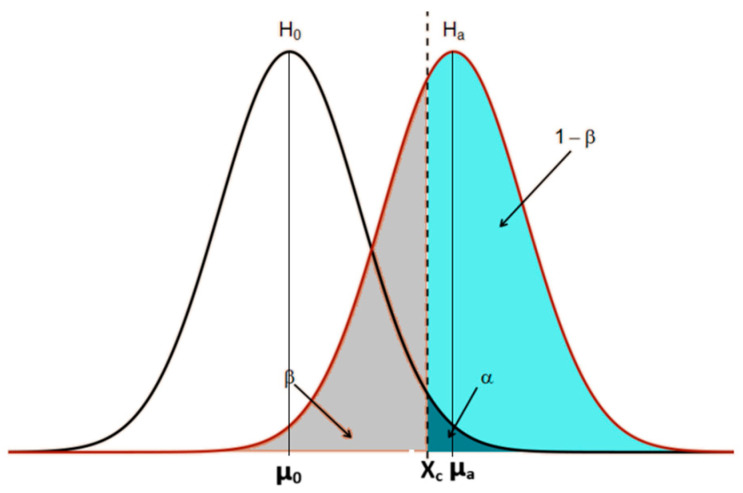
Representation of the histogram for two situations, H_0_ and H_a_, where one seeks to know with an error α how precisely the two measurements differ.

The test power was used to analyze the ability of the studied experiment to differentiate conditions with and without the sigma phase. An accurate estimation of the test power can predict the probability that a statistically significant difference will be detected based on a finite sample size under a real alternative hypothesis. If the power is too low, there is little chance of a significant difference being detected, and non-significant results are likely, even if there are, in fact, real differences. In Figure 2, (1 − β) corresponds to the test power. Values of (1 − β) greater than or equal to 1 (one) indicate a 100% or more difference and that the measurements will always differ. Values below 1 (one) indicate the probability of overlapping results. The value (1 − β) indicates the measurement system’s accuracy and corresponds to the test power in Figure 2.

In Figure 2, α is the error if the null hypothesis (H_0_) is rejected in favor of the alternative hypothesis (H_a_) when H_0_ is true. β is the error if H_0_ is not rejected when H_a_ is true. (1 − β) is the power of a hypothesis test, i.e., the probability of rejecting H_0_ when H_a_ is the true hypothesis. When (1 − β) is greater than or equal to 1 (one), it indicates a 100% or more difference and that the measurements will always differ.

The following equations are used to determine the test power:

(1) $${\bar{x}}_{c}=\mu_{0}+t_{c}\times \frac{S_{0}}{\sqrt{n}},$$

where ${\bar{x}}_{c}$ is the critical value of the H_0_, $S_{0}$ is the H_0_ standard error, $\mu_{0}$ is the H_0_ mean, n the number of measures, and $t_{c}$ the t Student for (n − 1) and α = 0.05, and

(2) $$Z_{\beta }=\frac{\left({\bar{x}}_{c}-\mu_{a}\right)\times \sqrt{n}}{S_{a}},$$

where $Z_{\beta }$ is the standard score, which represents how far from the mean a data point is, $\mu_{a}$ is the H_a_ mean, and $S_{a}$ is the H_a_ standard error.

The *Z_β_* value is then used to find the area of the region of (1 − β) shown in Figure 2, which represents how different H_0_ and H_a_ are, using a Z score table.

## 3. Results and Discussions

**Figure 3:**
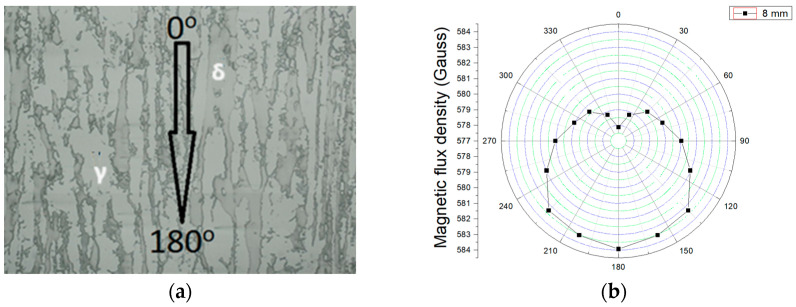
(**a**) The optical microscopy of the DSS structure in the as-received condition, where δ is the ferrite phase and γ the austenite, and (**b**) the magnetic flux density variation as a function of the rotation angle.

Figure 3a shows the optical microscopy of the as-received material, and Figure 3b depicts the polar plot of the measured magnetic flux density for the related stainless steel sample. Figure 1 shows that the easy magnetization direction is lamination (180°). This angle has the highest value of magnetic flux density, i.e., this direction corresponds to the one with the lowest magnetic losses. The present work was carried out with direct current and a fixed solenoid pole; therefore, under the experimental conditions, the polar graphs show the maximum at an angle of 0°. The plate was received in the hot rolled state, and all the measurements were performed in the rolled direction, corresponding to the easy magnetic direction.

In scanning electron microscopy, the angle formed between the X-ray source and the detector, i.e., the angle between the transmitted beam and the reflected beam, is usually designated as 2θ. In an experiment, the crystallographic plane cannot be observed; however, the transmitted and the reflected beam can be observed and, therefore, the 2θ can be experimentally assessed.

**Figure 4:**
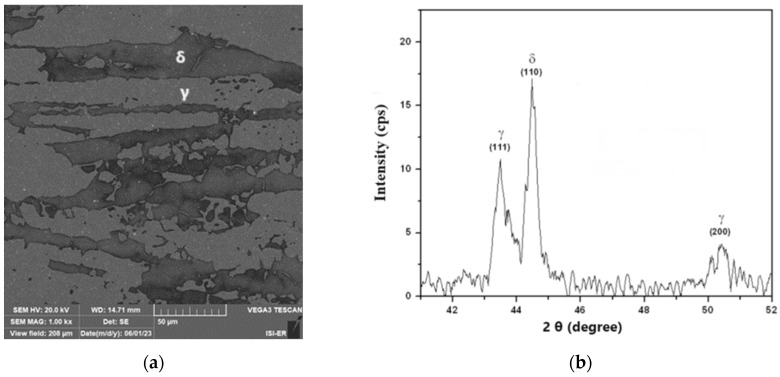
(**a**) The scanning electron microscopy image (10% KOH attack, 1000x of magnification) and (**b**) the X-ray diffractogram of the as-received condition sample (δ—ferrite and γ—austenite).

Figure 4a,b show the material’s scanning electron microscopy analysis results in the as-received condition and its X-ray diffraction. In Figure 4a, one can observe the presence of austenite islands, clear regions on a ferrite matrix, and dark regions, showing no formation of precipitates. The presence of these constituents is confirmed in the diffractogram of Figure 4b, where only peaks of austenite and ferrite can be seen. The ferrite constituent is ferromagnetic and the austenite paramagnetic. In the as-received condition, SAF 2205 steel has no ferrite matrix transformation. However, when heated at temperatures above 550 °C, precipitates are formed, including the sigma phase, which has a hardness of around 900 HV and, as it is rich in chromium, impoverishes the matrix of this element and compromises both the corrosion resistance and the tenacity of the material [1,2,3,4,8].

**Figure 5:**
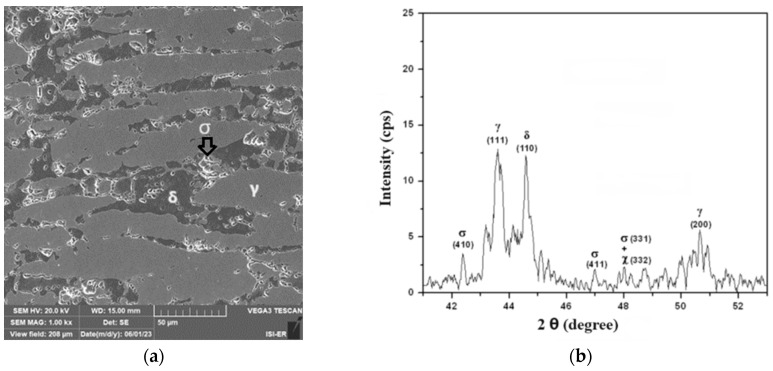
(**a**) The scanning electron microscopy image (10% KOH attack, 1000X of magnification) and (**b**) the X-ray diffractogram of the sample treated at 850 °C for ¼ h (δ—ferrite, γ—austenite, and σ—sigma).

Figure 5 presents the microscopy of the sample treated at 850 °C for ¼ h and its X-ray diffractogram. The presence of the sigma phase in the ferritic region can be observed as discontinuous precipitates formed from the ferrite grain boundaries. The austenite of duplex stainless steel does not transform, as the diffusion of atoms in it is about 100 times greater than in ferrite, which leads to concentrations occurring in this phase [1,2,3,10].

**Figure 6:**
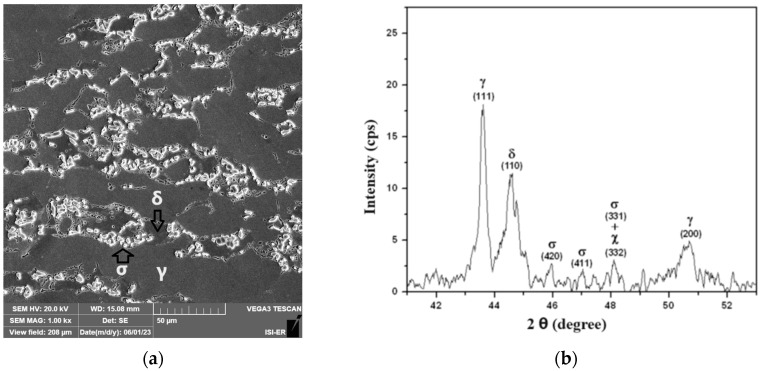
(**a**) The scanning electron microscopy image (10% KOH attack, 1000X of magnification) and (**b**) the X-ray diffractogram of the sample treated at 850 °C for 2 h (δ—ferrite, γ—austenite, and σ—sigma).

Figure 6 presents the microscopy of the sample treated at 850 °C for 2 h and its X-ray diffractogram. A greater presence of the sigma constituent can be observed in this case. The amount of the sigma phase was measured as being 5%, 17%, and 18% for the treatment times of ¼ h, 1 h, and 2 h, respectively. As the difference between the sample treated for 1 h and the one for 2 h was only 1%, Figure 6 presents only the scanning electron microscopy image and the X-ray diffractogram of the sample treated for 2 h.

The treatments with times of 1/4 h and 2 h show the presence of the chi phase. However, the χ phase forms before the precipitation of the σ phase and disappears once the σ phase starts to precipitate. The precipitation of the σ and χ phases decreases the magnetic properties of the steel because ferrite is ferromagnetic, and the χ and σ phases are paramagnetic phases. Electrolytic etching with 10% KOH solution preferably reveals the σ phase [1,33].

Ferrite microhardness measurements in the as-received, treated for ¼ h, and for 2 h conditions were carried out, being 215.15 (±13.4) for the as-received condition, 254.1 (±10.79) for the treated for ¼ h, and 292.5 (±10.4) for 2 h. Ten measurements were taken for each condition, and the confidence interval was obtained at 95%. The increase in hardness shows the formation of the sigma phase during treatment. The results of X-ray diffraction and scanning electron microscopy, combined with the microhardness, reinforce the understanding that there was no sigma formation before annealing, or if it did occur, the quantity was insufficient for detection with the techniques used. This indicates that the 5% sigma obtained is already enough to influence the results and that the effect of ferrite softening, if any, will not be a main factor in the present work.

**Figure 7:**
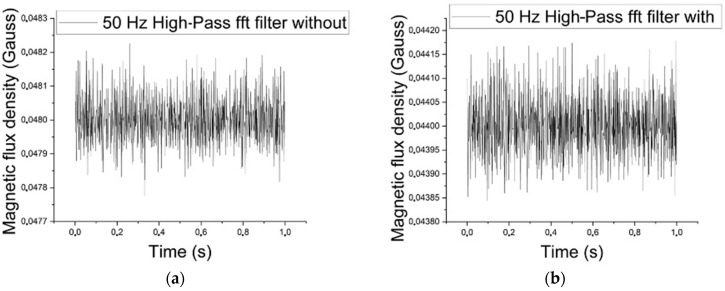
(**a**) Magnetic noise, measured in the magnetic flux density, of the conditions without the sigma phase, as a function of the time, after applying the 50 Hz high-pass filter for an excitation wave of 5 Hz and 1 V. (**b**) Magnetic noise of the conditions with 5% of the sigma phase, as a function of time, after applying the 50 Hz high-pass filter, for an excitation wave of 5 Hz and 1 V.

Figure 7a,b show the magnetic noise of the conditions without the sigma phase and with 5% of it, respectively, after applying the 50 Hz high-pass filter for an excitation wave of 5 Hz frequency and 1 V amplitude. The presence of sigma precipitates acts as anchor points for the movement of the magnetic domain walls and reduces the magnetic flux density value [2,3]. Also, there is the contribution of the paramagnetism of the sigma phase [1,5]. The RMS values for the conditions without and with the sigma phase were determined as equal to 0.04825 and 0.04437 gauss, respectively. The test power was calculated to be around 6, indicating a more than 100% certainty in differentiating between the two cases despite only having a 10% difference. Although the values obtained are small in magnitude, measurements in different conditions can differentiate the two cases without overlapping measurements, thus confirming accuracy.

**Figure 8:**
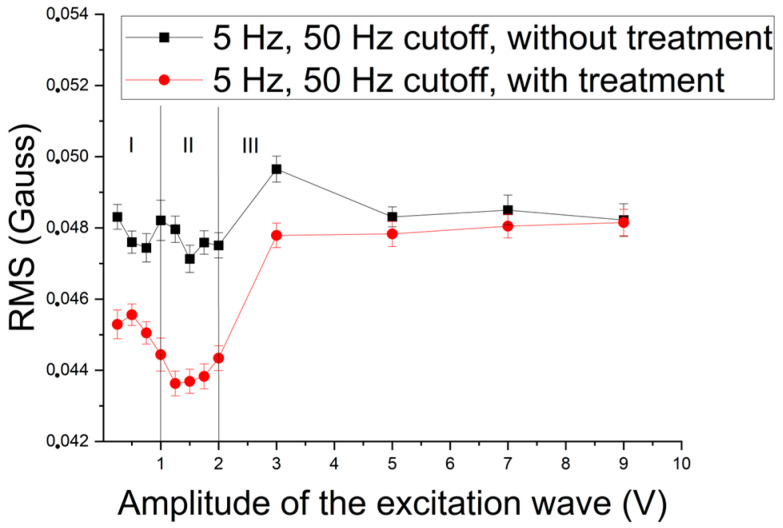
RMS of the signal acquired by the Hall sensor as a function of the amplitude of a 5 Hz excitation wave applied in samples with and without treatment and a cutoff of 50 Hz.

Next, this study concerned itself with the characteristics of the excitation wave able to detect the presence of the sigma phase by magnetic noise analysis in the samples under study, with the application of excitation waves with a frequency of 5 Hz and cutoff frequencies of 50 Hz and 250 Hz. Figure 8 shows the RMS variation in the signal acquired by the Hall sensor as a function of the amplitude of the excitation wave applied to the samples without precipitate and treated at a temperature of 850 °C for ¼ h, for an excitation frequency of 5 Hz and amplitudes from 0.25 V to 9 V, and a cutoff frequency of 50 Hz.

Analyzing Figure 8, one can perceive the existence of three distinct regions, named I, II, and III, one for 0.25 V to 1 V amplitudes, another for 1 V to 2 V amplitudes, and the last one for 3 V to 9 V amplitudes. This figure depicts the RMS variation as a function of the amplitude of the excitation wave for the samples as received and with 5% of the paramagnetic sigma constituent. The curve for the sample with the presence of the sigma constituent is shifted downwards due to the reduction in its ferromagnetism, with the transformation of part of the ferrite constituent into sigma precipitates. In region I, there is also a reduction in RMS values due to blocking the movement of the walls of the magnetic domains due to the presence of sigma precipitates. The increased magnetic flux in region II leads to a greater detection of these precipitates and their contribution to the blockage. However, in the third region (III), as there is a large increase in magnetic flux, there is an increase in the noise generated by the movement of the magnetic domain walls, which try to overcome the blocking effect of the sigma constituent. Similar behavior was observed in [2,3,10]. The logarithmic scale was chosen for the amplitude to facilitate the visualization of the regions in the presented graphs.

A point was chosen in each region of Figure 8 to analyze the test power of the measurements, that is, their ability to differentiate the two conditions. The test power was applied to the waves with 0.25 V, 1.25 V, and 7 V of excitation amplitude, and results of 4.04, 6.26, and 0.6, respectively, were obtained. The value of 0.6 indicates that in the third region for this amplitude, there is a 60% overlap of the results of the two conditions. However, for the first and second regions, the values were above 1 (one), indicating with total certainty that the technique accurately differentiates the two conditions. The values of 4.04 and 6.26 indicate the sensitivity of the measurement setup.

**Figure 9:**
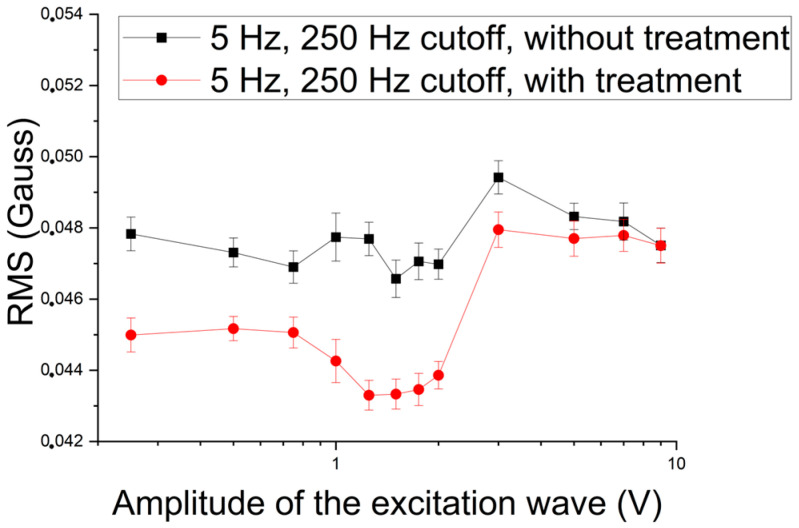
RMS of the signal acquired by the Hall sensor as a function of the amplitude of the 5 Hz excitation wave applied in samples with and without treatment and a cutoff frequency of 250 Hz.

Figure 9 shows the RMS variation of the signal acquired by the Hall sensor as a function of the amplitude for an excitation frequency of 5 Hz and a cutoff frequency of 250 Hz, applied in the samples with and without treatment. The same regions identified in the previous case can be observed in Figure 9. It can also be noted that the RMS values of the treated sample signal are lower than those of the untreated sample. This can occur due to the paramagnetism of the sigma phase, which reduces the material’s permeability [2,3], or to the movement of magnetic domain walls’ blocking because of this newly formed structure [2,13,34].

**Figure 10:**
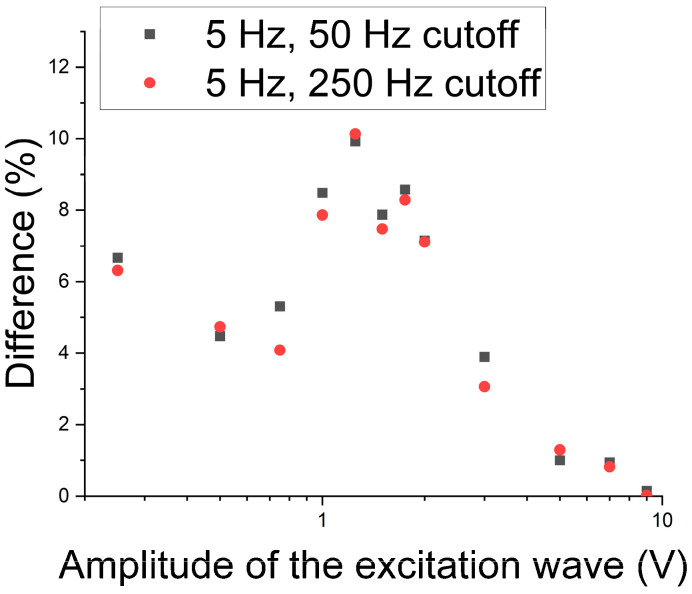
Module of the difference in RMS as a function of the amplitude of the 5 Hz excitation wave and the two tested cutoff frequencies.

To identify the best working region for the 5 Hz excitation wave, a graph was built concerning the module of the difference in the RMS of the two cases, without and with the presence of the sigma phase in a percentage, as shown in Figure 10. It is noted that the greater differences between the two cases in the central region of the graph are the most evident for the performed test in detecting the sigma phase. This region corresponds to excitation wave amplitudes ranging from 1 V to 2 V.

**Figure 11:**
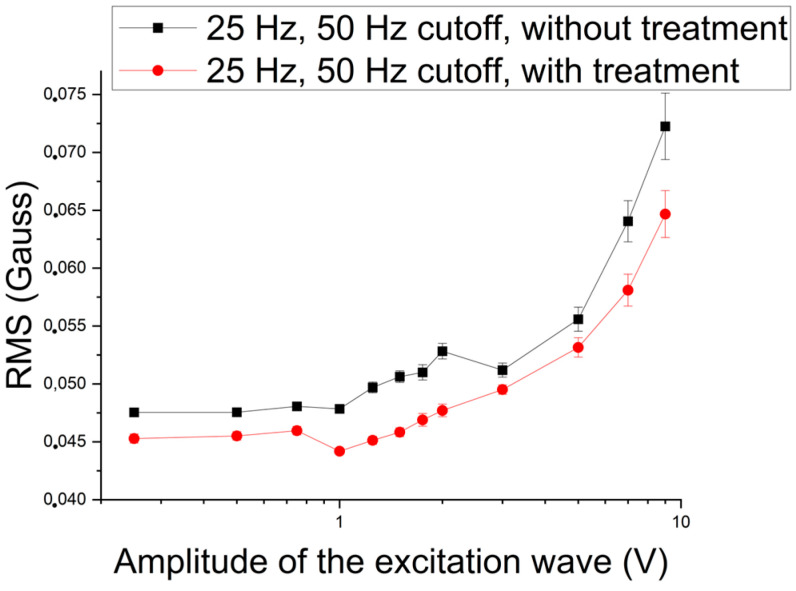
RMS of the signal acquired by the Hall sensor as a function of the amplitude of the excitation wave applied to the samples with and without treatment for an excitation frequency of 25 Hz and a cutoff of 50 Hz.

**Figure 12:**
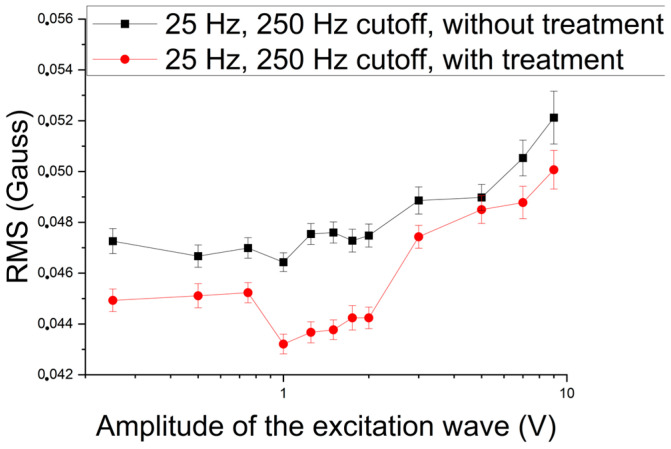
RMS of the signal acquired by the Hall sensor as a function of the amplitude of the excitation wave applied to the 8 mm samples with and without treatment with an excitation frequency of 25 Hz and a cutoff of 250 Hz.

Next, the influence of applying 25 Hz sinusoidal waves was studied. Figure 11 and Figure 12 show the RMS values as a function of the amplitude of the excitation wave with 50 Hz and 250 Hz cutoffs. Figure 11 shows the influence of the 50 Hz cutoff, where the three regions detected when applying the 5 Hz wave can be observed. The first stage presents behavior in the conditions of with and without precipitate in a parallel way, followed by a fall, but with a gradual increase from the second region. This means that the harmonics of the excitation wave begin to influence the RMS values in the noise region, indicating that the increase in frequency and the application of cutoffs in the lower-frequency region lead the harmonics of the excitation wave to influence the noise region and increase its amplitude.

In Figure 12, one can see that the first region is still quite well-defined. The second stage goes from 1 V to 2 V, and a gradual increase is observed due to the increase in frequency, leading the harmonics to interference in this region. However, for these, the behavior was like that of the 5 Hz excitation waves.

**Figure 13:**
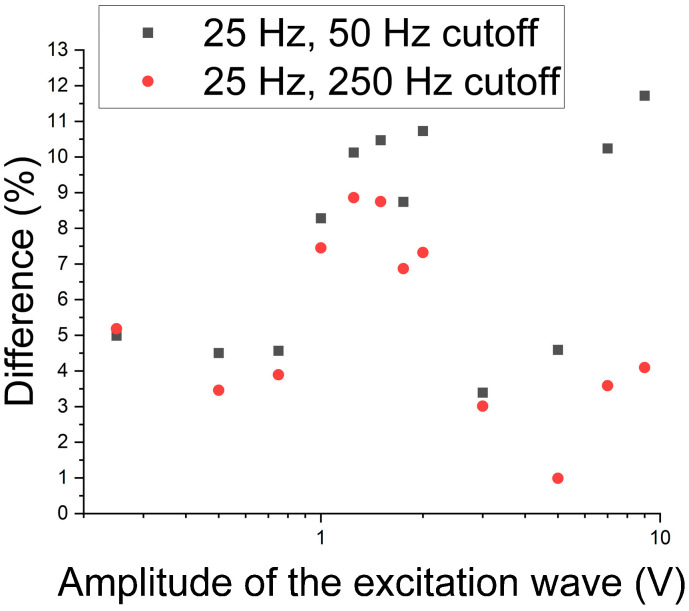
RMS difference module as a function of the wave amplitude of the applied 25 Hz transmitter wave and the two cutoff frequencies.

Figure 13 shows the module of the difference in the RMS as a function of the wave amplitude to determine which regions lead to the best values that differentiate the percentage as a function of the amplitude of the 25 Hz excitation wave. One can perceive that in the region from 1 V to 2 V, the best results were found for the cutoff frequency of 25 Hz. For the 50 Hz cutoff, there appear points with greater values for the amplitudes of waves of 7 V and 9 V, where one can perceive that as the cutoff is reduced to 50 Hz and the frequency is increased, these points are also more distinguished. These are due to the harmonic of the main wave interfering in this region.

**Figure 14:**
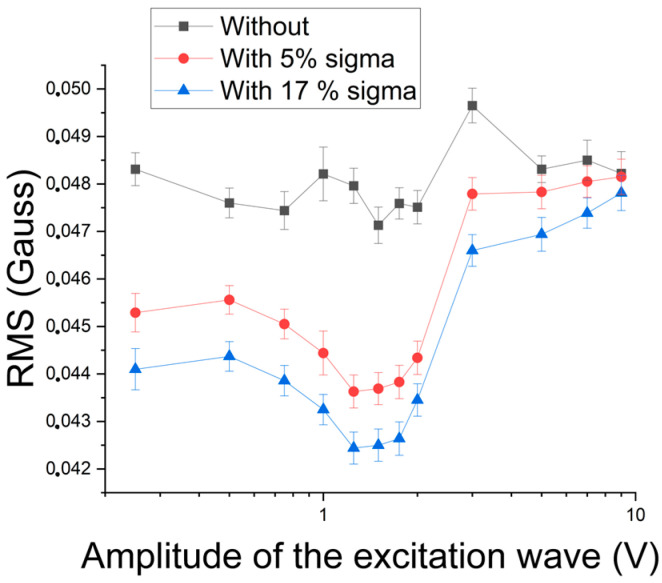
RMS of the signal acquired by the Hall sensor as a function of the amplitude of the 5 Hz excitation wave applied to 8 mm samples for conditions with different amounts of the sigma phase and a cutoff of 50 Hz.

Figure 14 shows the RMS variation as a function of the excitation amplitude for waves of 5 Hz and 1 V regarding the amount of the sigma constituent. The excitation wave was chosen to be in the region with the best results. Thus, the magnetic noise obtained by the methodology can follow the formation of the sigma phase.

**Figure 15:**
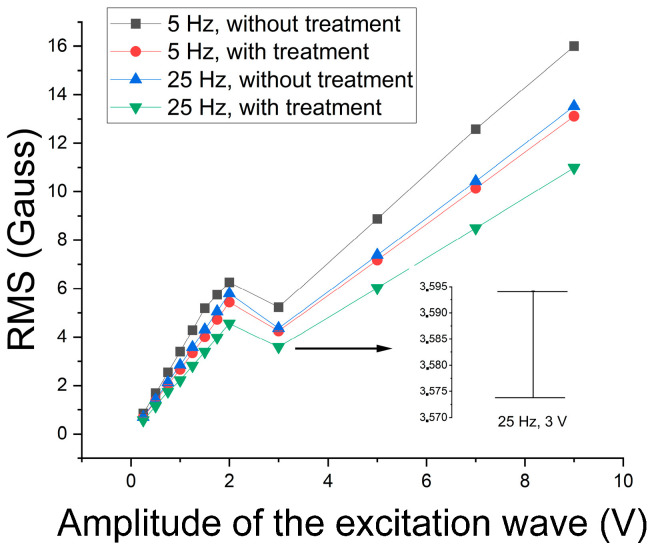
RMS variation of the first harmonic as a function of the amplitude of the 5 Hz and 25 Hz excitation waves and samples with and without the sigma phase. The 25 Hz and 3 V excitation wave error range is shown.

Next, the ability to detect the constituent sigma’s presence was studied by analyzing the excitation wave’s harmonics [18,19,20]. Figure 15 shows the variation in the RMS of the first harmonic of 5 Hz and 25 Hz excitation waves as a function of their amplitude. A similar behavior could be observed between the studied frequencies: an increase to the amplitude of 1 V, followed by a decrease to 2 V, and a linear growth again. In addition, the signals with precipitates present lower values than those without the sigma constituent. The lower values, obtained with precipitates, must combine the paramagnetism of the sigma constituent, formed from the ferromagnetic ferrite, and the blocking of the magnetic domain walls’ movement by forming coarse precipitates. This type of behavior has been observed in the literature [2,3]. However, the drop in values from 2 V, as occurred for both conditions, indicates that the increase in amplitude leads to the detection of the contribution of paramagnetic austenite with the increase in magnetic flux [10]. It can also be noted in Figure 15 that the RMS values fall with increasing frequency. This was expected, as the penetration depth has an inverse behavior to the increase in frequency due to the surface effect [12]. Because the error range of the measurements is too small to be easily readable, a magnification showing the range detail for the sample with thermal treatment and the 25 Hz and 3 V excitation wave is visible in Figure 15.

**Figure 16:**
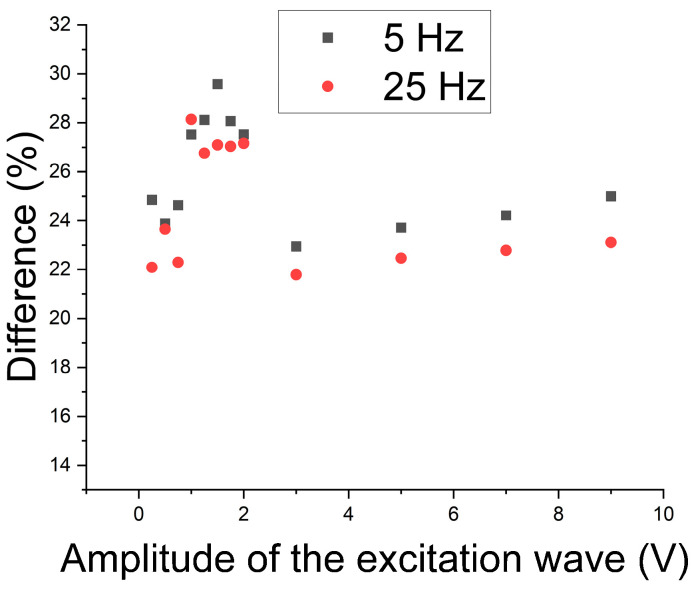
Differences in percentage between conditions without and with the sigma phase as a function of the amplitude of the 5 Hz and 25 Hz excitation waves.

To determine the best working region, a graph was built of the difference between the conditions without and with precipitate in a percentage as a function of the amplitude of the 5 Hz and 25 Hz excitation waves (Figure 16). One can note that the best region continues to be from 1 V to 2 V. Still, the range of difference in this region with magnetic noise analysis was around 7% to 10% before, and now it becomes from 25% to 32% when the amplitude of the first harmonic is analyzed. Thus, the first harmonic was applied to monitor the formation of the sigma constituent.

**Figure 17:**
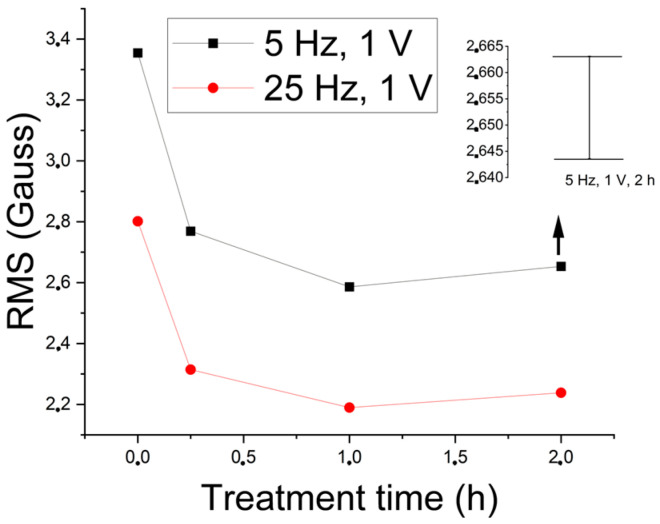
RMS variation of the first harmonic as a function of the treatment time for the 5 Hz and 25 Hz excitation waves. The 5 Hz and 1 V excitation wave error range is shown.

Next, a study concerning the follow-up of the formation of the sigma constituent was carried out. Considering that in the range from 1 V to 2 V, the increase in the magnetic flux density begins to detect the paramagnetic austenite, the study was conducted for 1 V, which takes values still in the region of better results for detecting the sigma phase. Figure 17 shows the RMS variation of the first harmonic of the excitation wave as a function of the treatment time at 850 °C for an amplitude of 1 V and frequencies of 5 Hz and 25 Hz. Figure 17 shows a drop in RMS values to a plateau after 1 (one) hour of treatment. The test was shown to be sensitive to the detection of 5% of the sigma phase, which is harmful to the tenacity of the material and occurs after 15 min of treatment. The plateau is due to the amount of formed constituent being close to the treatment times of 1 h and 2 h.

A correlation between the absorbed energy per impact and the amount of the sigma phase for the same studied plate showed an absorbed energy of 76.67 J for the as-received condition, 13.87 J for the amount of 5% of the sigma phase, and 10.2 J for the amounts of 17% and 18% of the sigma phase [2]; that is, there is a quick drop to 5% and then a stabilization around 10 J. The values of 5%, 17%, and 18% correspond to the amounts of the sigma phase for the treatment times of ¼ h, 1 h, and 2 h, respectively. This indicates that the variation in the first harmonic RMS directly correlates with the variation in energy absorbed by impact and serves as a parameter to follow the formation of the harmful sigma phase in the studied stainless steel. Correlations between the amount of the sigma phase and the energy absorbed per impact have shown the same behavior in the literature [1,35].

In the present work, a study of detecting the formation of sigma precipitates was carried out through an electromagnetic test with the replacement of the pick-up coil by a magnetic field sensor. The magnetic noise and the first harmonic amplitude variation for detecting harmful sigma precipitate were analyzed. One can note that both parameters detect the constituent studied, and the best results were obtained with the harmonic analysis. It was also verified that the best test amplitude of the excitation wave to detect the presence of the sigma phase, without interference from paramagnetic austenite, is 1 V for frequencies of 5 Hz and 25 Hz.

## 4. Conclusions

In this work, a study was carried out on applying magnetic noise and the amplitude of the first harmonic of the excitation wave to detect the formation of the harmful constituent sigma in duplex stainless steel. An electromagnetic test was applied with the replacement of the pick-up coil by a magnetic field sensor. The following conclusions were obtained:

- The applied electromagnetic test detected the presence of the sigma phase, with the replacement of the pick-up coil by the Hall effect sensor.
- The RMS values of the samples with the presence of the sigma phase were lower than those of the condition without precipitate, and the graphs of the RMS values as a function of the amplitude of the excitation wave showed three regions with different behaviors.
- The reduction in the RMS values in the first region occurred probably due to the movement of magnetic domain walls being blocked by the presence of the sigma phase. On the other hand, the increase in the magnetic flux in region II led to a greater detection of the phase precipitates and their contribution to the blockage. However, for region III, there was an increase in noise generated by the movement of the walls, which tried to overcome the blocking effect of the sigma constituent.
- Applying waves with the studied frequencies detected the presence of the sigma constituent, with the best results being for excitation waves with amplitudes from 1 V to 2 V, both by magnetic noise and the amplitude of the first harmonic analysis.
- The analysis, both by magnetic noise and the amplitude of the first harmonic, showed the presence of a plateau between 1 V and 2 V, which was attributed to the increase in the magnetic flux becoming influenced by the presence of paramagnetic austenite.
- The best results for detecting the sigma phase were obtained by analyzing the amplitude of the first harmonic, which was used to follow the constituent sigma formation and proved its effectiveness.

## Data Availability

The data supporting this study’s findings are available from the first author, João Silva, upon reasonable request.
